# Biophysical models accurately characterize the thermal energetics of a small invasive passerine bird

**DOI:** 10.1016/j.isci.2023.107743

**Published:** 2023-08-26

**Authors:** Marina Sentís, Cesare Pacioni, Annelies De Cuyper, Geert P.J. Janssens, Luc Lens, Diederik Strubbe

**Affiliations:** 1 Terrestrial Ecology Unit, Department of Biology, Faculty of Sciences, Ghent University, 9000 Ghent, Belgium; 2 Department of Veterinary and Biosciences, Faculty of Veterinary Medicine, Ghent University, 9820 Merelbeke, Belgium

**Keywords:** Biological sciences, Biophysics, Natural sciences

## Abstract

Effective management of invasive species requires accurate predictions of their invasion potential in different environments. By considering species’ physiological tolerances and requirements, biophysical mechanistic models can potentially deliver accurate predictions of where introduced species are likely to establish. Here, we evaluate biophysical model predictions of energy use by comparing them to experimentally obtained energy expenditure (EE) and thermoneutral zones (TNZs) for the common waxbill *Estrilda astrild*, a small-bodied avian invader. We show that biophysical models accurately predict TNZ and EE and that they perform better than traditional time-energy budget methods. Sensitivity analyses indicate that body temperature, metabolic rate, and feather characteristics were the most influential traits affecting model accuracy. This evaluation of common waxbill energetics represents a crucial step toward improved parameterization of biophysical models, eventually enabling accurate predictions of invasion risk for small (sub)tropical passerines.

## Highlights

• Nichemapper accurately predicts common waxbill’s energy use and thermoneutral zone
• Body temperature, metabolic rate, and feather properties affect model performance
• The degree of ptiloerection influences model accuracy
• These models outperform traditional time-energy budget methods

## Graphical abstract

**Figure:**
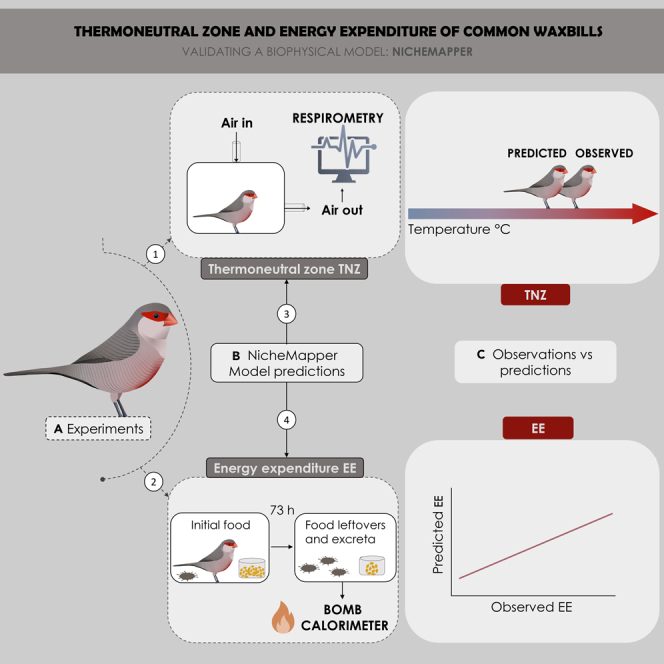

## Introduction

Human activities impact global biodiversity in different ways, e.g., by decreasing population sizes, shifting geographic distribution ranges, or changing species compositions.^1^^,^^2^^,^^3^ For example, rising temperatures, largely caused by burning of fossil fuels and land-use change, have increased the extinction risk of many species,^4^ and introductions of exotic species have become a major threat to native species worldwide.^5^ To ensure effective conservation strategies and evaluate different management scenarios, it is imperative to accurately predict how species will respond to such rapid and diverse environmental changes.^6^ Tools to detect and prevent non-native species imports, protect critical habitats for threatened species, and identify areas of high conservation value to be added to networks of protected areas are therefore urgently needed.^7^ Among the most widely used approaches to anticipate environmental changes are correlative distribution models.^8^ Such “species distribution models” (SDMs) link species occurrence data with the environmental conditions at their location to characterize species’ ecological niches and allow to identify geographical areas suitable for species’ establishment and persistence.^9^^,^^10^^,^^11^ SDMs have the advantage that they do not require detailed knowledge of species ecology, and estimates of suitable areas can be obtained relatively easily for a wide range of organisms. SDMs have therefore become a fundamental tool in ecology and biodiversity management, e.g., for informing management schemes for threatened species, identifying areas potentially suitable for species’ translocations, or guiding surveys aimed at locating extant populations of species of conservation concern.^12^^,^^13^

SDM applications to global change phenomena such as climate change or biological invasions, while prevalent, are more controversial as they require extrapolation into novel environments, i.e., outside the range of conditions on which the models were trained.^14^^,^^15^^,^^16^^,^^17^ Therefore, alternative frameworks for predicting species’ responses to novel conditions have more recently come to the fore. These “process-based” models combine environmental conditions with biological processes, such as growth, reproduction, and dispersal, to assess which climates can be tolerated by the species of interest.^18^ Such an approach does not rely on contemporary species distributions as input data, and it is claimed that it can more accurately predict species’ responses beyond current conditions.^19^^,^^20^ A main class of process-based models are mechanistic, biophysical models that calculate key organismal heat and mass balances based on biophysical first principles.^21^ Biophysical models link behavioral, morphological, and physiological traits with spatial environmental data to determine the energetic requirements of species.^21^^,^^22^ This allows the investigation of how different environmental factors (such as climate, weather, and habitat) and functional traits (i.e., characteristics, such as body shape or feather/fur properties, that directly impact survival, development, growth, and reproduction)^23^ may affect the potential distribution of species.^18^^,^^24^ For example, when modeling the impacts of climate change on the American pika (*Ochotona princeps*), Mathewson et al.^25^ showed that biophysical models that account for behavioral thermoregulation predicted less loss of suitable habitat compared with correlative SDMs.

NicheMapper (NM), the most complete biophysical modeling framework available to date,^21^^,^^26^ combines information on species’ morphology, ecophysiology, and behavior to determine animal energy expenditure (EE) given the local climate and weather. NM defines and models EE as the sum of the energy used by an organism for various processes, including basal metabolism, thermoregulation, digestion, and physical activity. At temperatures outside the thermoneutral zone (TNZ, the temperature range where an endotherm can maintain its body temperature without elevating its metabolic rate beyond the basal level), NM calculates the metabolic rate that an individual must maintain to remain in homeothermy. When, under cold stress, the calculated rate is higher than the maximum metabolic rate, the individual is assumed to die from hypothermia. When the only model solution for homeothermy under heat stress is a predicted metabolic rate lower than the basal (i.e., minimum) metabolic rate, the individual is assumed to die of hyperthermia.^24^ Since the balance between energy acquisition and expenditure is essential for growth, survival, and reproduction, EE is an important factor in understanding animal ecology and evolution.^27^^,^^28^ Moreover, the ability to predict EE at temperatures outside species’ TNZ may allow for a better estimation of how animals are likely to respond to a changing environment. For example, global warming may cause some species to increase EE to keep their body temperature constant as ambient temperatures progressively deviate from their TNZ, ultimately reducing their fitness.^29^^,^^30^^,^^31^^,^^32^ This effect can be observed, for instance, in passerines, which rely on panting as their primary pathway for evaporative heat dissipation, incurring a significant metabolic cost.^33^ Understanding and predicting how species’ EE may respond to climate change can thus inform conservation planning, e.g., by identifying species likely to be most strongly affected.^34^ However, compared to the widespread use of SDM, the use of biophysical models as a tool for ecological forecasting is still lagging behind.^35^ This is likely because these models require an accurate biological understanding of the species of interest and its relationship with the environment, as a large number of ecophysiological parameters need to be known for correct parameterization.^15^ The difficulty in obtaining reliable estimates for key functional traits, and the sensitivity of the model to variations in parameter estimates, have been cited as the main sources of uncertainty and error in biophysical model predictions.^9^ Moreover, the successful implementation of these models may necessitate extensive knowledge of biophysical and biochemical principles. As a result, their effective implementation calls for expertise and involves a challenging learning process.^20^

To date, NM has been successfully used to predict contemporary range distributions and climate effects in a variety of bird species,^36^^,^^37^^,^^38^ but only a few studies have critically evaluated its ability to accurately characterize TNZs and associated EE (i.e., studies on Hawaiian honeycreepers [*Hemignathus virens*, *H. parvus*^38^], Brünnich’s guillemots and little auks [*Uria lomvia*, *Alle alle*^39^], double-crested cormorants [*Phalacrocorax auritus*^40^^,^^41^], whooping cranes [*Grus americana*^42^], and budgerigar [*Melopsittacus undulatus*^36^]). More recently, NM was shown to accurately model thermoregulatory responses to heat for three arid-zone bird species: southern yellow-billed hornbill (*Tockus leucomelas*), southern pied babbler (*Turdoides bicolor*), and southern fiscal (*Lanius collaris*).^43^ However, how NM performs in assessing the thermoregulation of small-bodied passerine birds remains virtually unstudied. Smaller birds have a relatively higher surface-to-volume ratio than larger ones and thus radiate more body heat per unit of mass. Given these higher thermoregulatory losses, smaller birds also require more nutrients and energy at a faster rate than larger birds and are less well equipped than the latter to cope with lower temperatures.^44^ As avian body masses are strongly right skewed,^45^ most birds are characterized by body masses lower than those of the aforementioned species.

To bridge this knowledge gap, here we assess the ability of NM to simulate the TNZ and EE of a small sub-Saharan estrildid finch, the common waxbill (*Estrilda astrild*, 7–9 g). This species has become invasive in many regions of the world^46^ and occupies colder areas than its native range.^47^ Evaluating and testing the ability of NM to simulate the thermal and energetic properties of common waxbills are a first step toward a more correct model parameterization for obtaining mechanistic forecasts of the invasion risk of small (sub)tropical passerines, which are well represented among the world’s successful invasive bird species.^48^^,^^49^ Moreover, while available NM studies (see above) compare mechanistic model predictions with data gleaned from other populations or even species, here we directly measure the TNZ and EE on a single set of captive individuals. First, we parameterize NM using functional trait data measured on 14 captive common waxbills and museum specimens. Second, we obtain predicted TNZ by simulating a metabolic chamber, which we then compare to the empirically measured TNZ through respirometry experiments. Third, we provide NM with the range of ambient temperatures to which our waxbills were exposed during a 73-h experiment to obtain a predicted EE, which we compare with empirical estimates of net energy intake (NEI), obtained by bomb calorimetry of consumed food and excreta. Fourth, we compare the obtained NEI with values estimated from classical time-energy budget approaches, where the duration of daily activities (such as foraging and resting) is multiplied by estimated energetic costs to obtain total daily EE.^27^ For these comparisons, we take into account differences in body mass between the start and the end of the 73-h study period as NEI can only be considered equal to EE only when animals maintain constant body mass.^50^ For both TNZ and EE predictions, we employ a scenario-based approach that considers different degrees of ptiloerection (a behavioral thermoregulatory response whereby birds fluff their feathers to trap air to increase insulation and decrease heat loss), based on estimates found in the literature.^37^^,^^38^^,^^51^ Finally, we carry out a sensitivity analysis to identify which parameters are most influential in the model predictions of TNZ and EE of common waxbills. To the best of our knowledge, this is one of the first assessments of NM’s ability to model the thermal energetics of a temperature-sensitive and high-energy-demanding bird species.

## Results

Information on metabolic rates was obtained through flow-through respirometry. Daytime resting metabolic rates (RMRs) amounted to 0.37 ± 0.04 W, compared to night-time (i.e., basal) metabolic rates (BMRs) of 0.20 ± 0.05 W in summer and 0.17 ± 0.03 W in autumn. Both (log) BMR and (log) RMR were positively correlated with (log) body mass (p ≤ 0.01). Common waxbills’ food intake during the 73-h trial was on average 8.06 ± 1.88 g of hulled millet, and the mean amount of excreta produced during that period was 1.41 ± 1.59 g. The mean gross energy content of the food consumed was 151.84 ± 35.56 kJ, and the average gross energy content of the excreta was 20 ± 23.72 kJ, resulting in an average NEI of 109.4 ± 15.5 kJ. Mean body mass after the experiment was 7.99 ± 0.93 g, and mean change in body mass was 0.19 ± 0.35 g.

**Figure 1:**
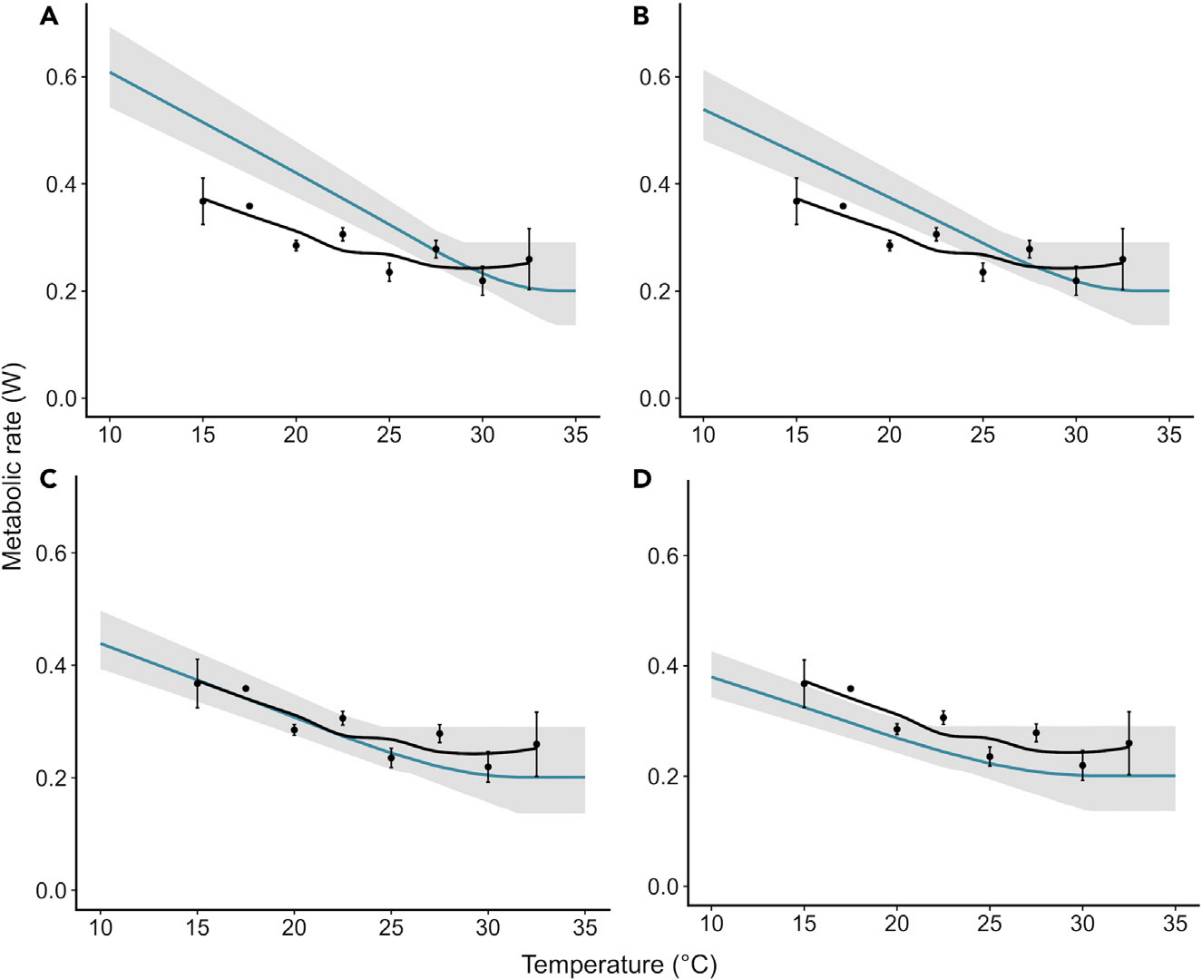
Predicted vs. recorded metabolic rate (W) of common waxbills at different ambient temperatures (°C) using different degrees of ptiloerection (A–D) (A) Increase in feather depth of 35%,^51^ B) increase in feather depth of 75%,^37^ C) increase in feather depth of 200%,^38^ D) full increase in feather depth, where feather depth equals feather length. The blue line represents the predicted metabolic rate per temperature, and the shaded region shows the 90% confidence interval. The black points and line depict the recorded metabolic rate, and the error bars represent the standard error.

**Figure 2:**
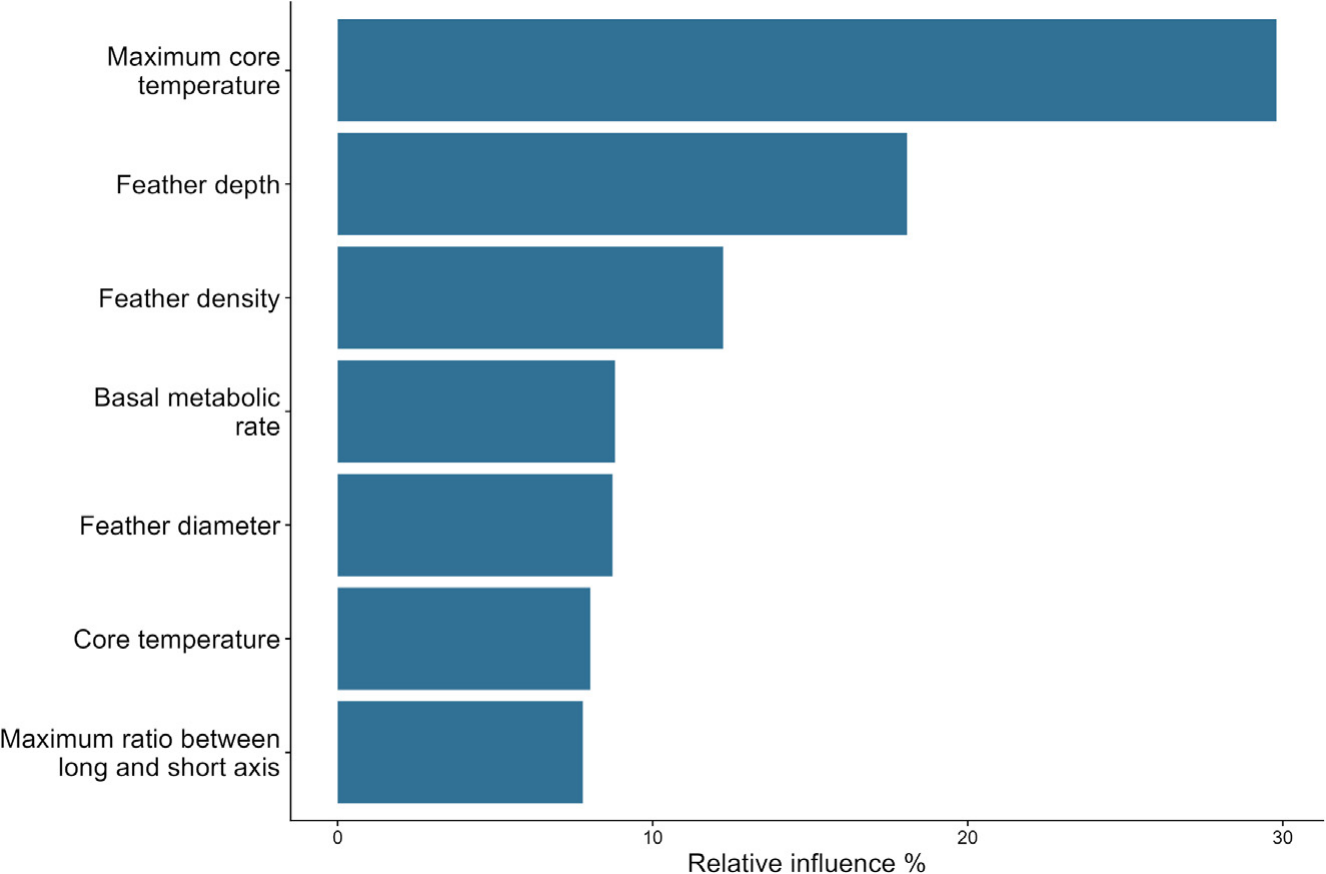
Relative influence of the most important parameters in predicting the lower bound of the thermoneutral zone (TNZ)

The four scenario-based mechanistic model predictions of the TNZ showed the best match between the predicted and recorded TNZ when allowing ptiloerection to increase to three times (200%) the measured feather layer depth, from 2.0 to 6.0 mm, with a root-mean-square error (RMSE) value (a commonly used metric for measuring the accuracy of predictive models) of 0.034. This degree of ptiloerection corresponds to ∼40% of the maximum feather depth that would be achieved by full ptiloerection (i.e., assuming feathers can be erected fully perpendicular to the body surface so that the depth of the feather layer equals the length of the feathers). Such full ptiloerection resulted in a comparatively higher RMSE value of 0.048. Other scenarios resulted in an even lower model fit, with RMSE equal to 0.058 when allowing a 75% increase and 0.092 for a 35% increase (Figure 1). A sensitivity analysis (Table 1) showed that predictions of the lower bound of the TNZ were most strongly influenced by maximum body temperature and feather depth (variable importance of 29.8% and 18.1%, respectively), followed by feather density (importance of 12.2%), BMR, feather diameter, and core body temperature (importance of ∼8% each), and lastly body shape (i.e., the maximum allowed ratio of body length to width, importance of 7.8%) (Figure 2).

**Table 1:** Morphological and physiological parameters used in the sensitivity analyses

Parameter	Minimum value	Maximum value
Body temperature, TC (°C)	39	42
Max body temperature, TC_MAX (°C)	39	44
Max ratio between long and short axis, SHAPE_B_MAX	1.2	5
Feather diameter dorsal, DHAIR (mm)^a^	0.019	0.11
Feather length, LHAIR (mm)	7.6	25
Feather depth, ZFUR (mm)	2.17	16.11
Feather density, RHO (1/mm^2^)^b^	0.74	140
Basal metabolic rate, QBASAL (W)	0.15	0.26
Body mass, AMASS (g)	6	10

a Minimum value Fitzpatrick et al.^42^ and maximum value Deville et al.^74^

b Minimum value Deville et al.^82^ and maximum value Fitzpatrick et al.^42^

**Figure 3:**
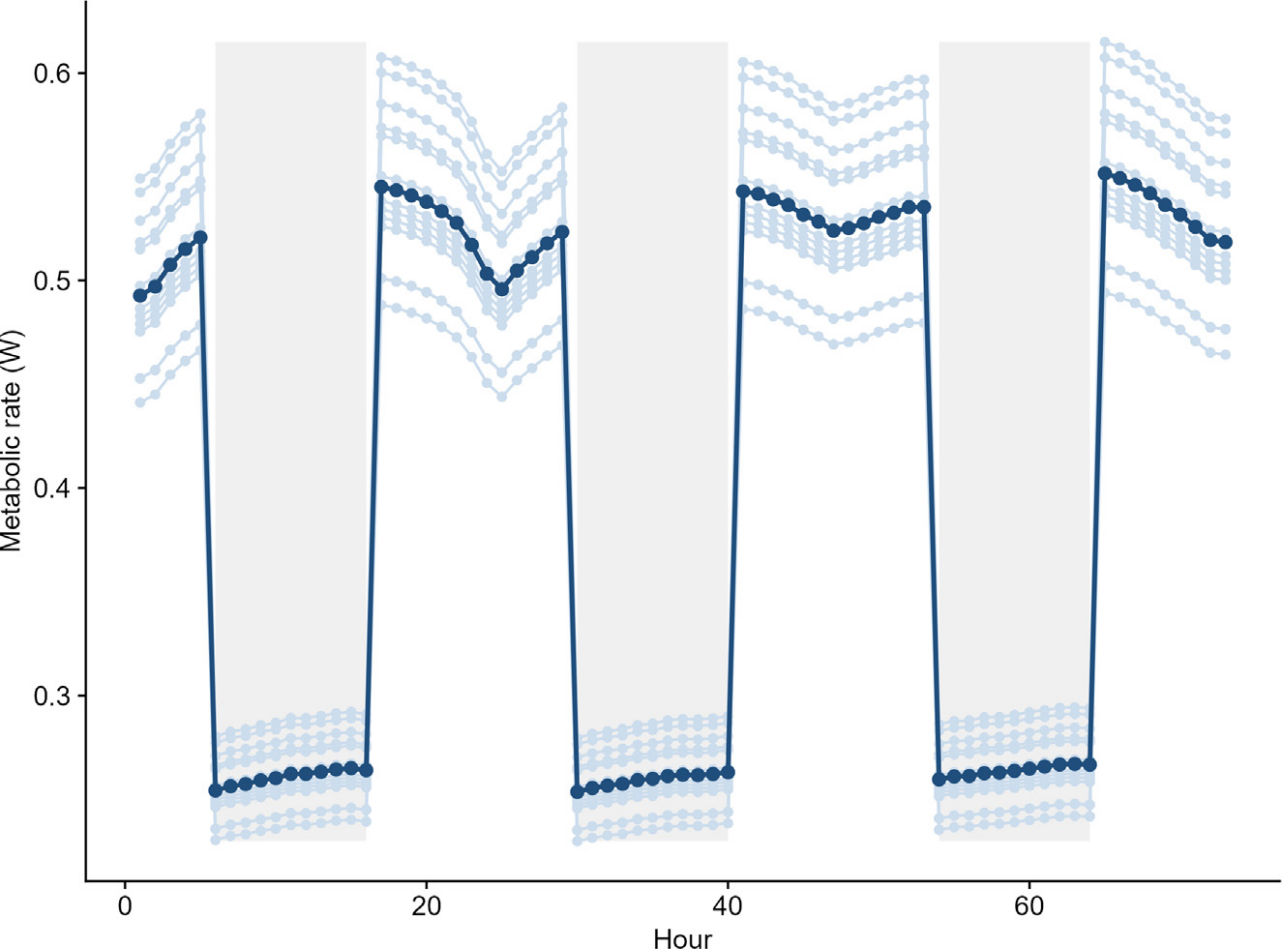
Predicted hourly metabolic rate (W) for a 73-h period The light-blue line and dots represent the metabolic rate of each individual, and the dark blue represents the average individual. Night times are depicted as shaded areas.

**Figure 4:**
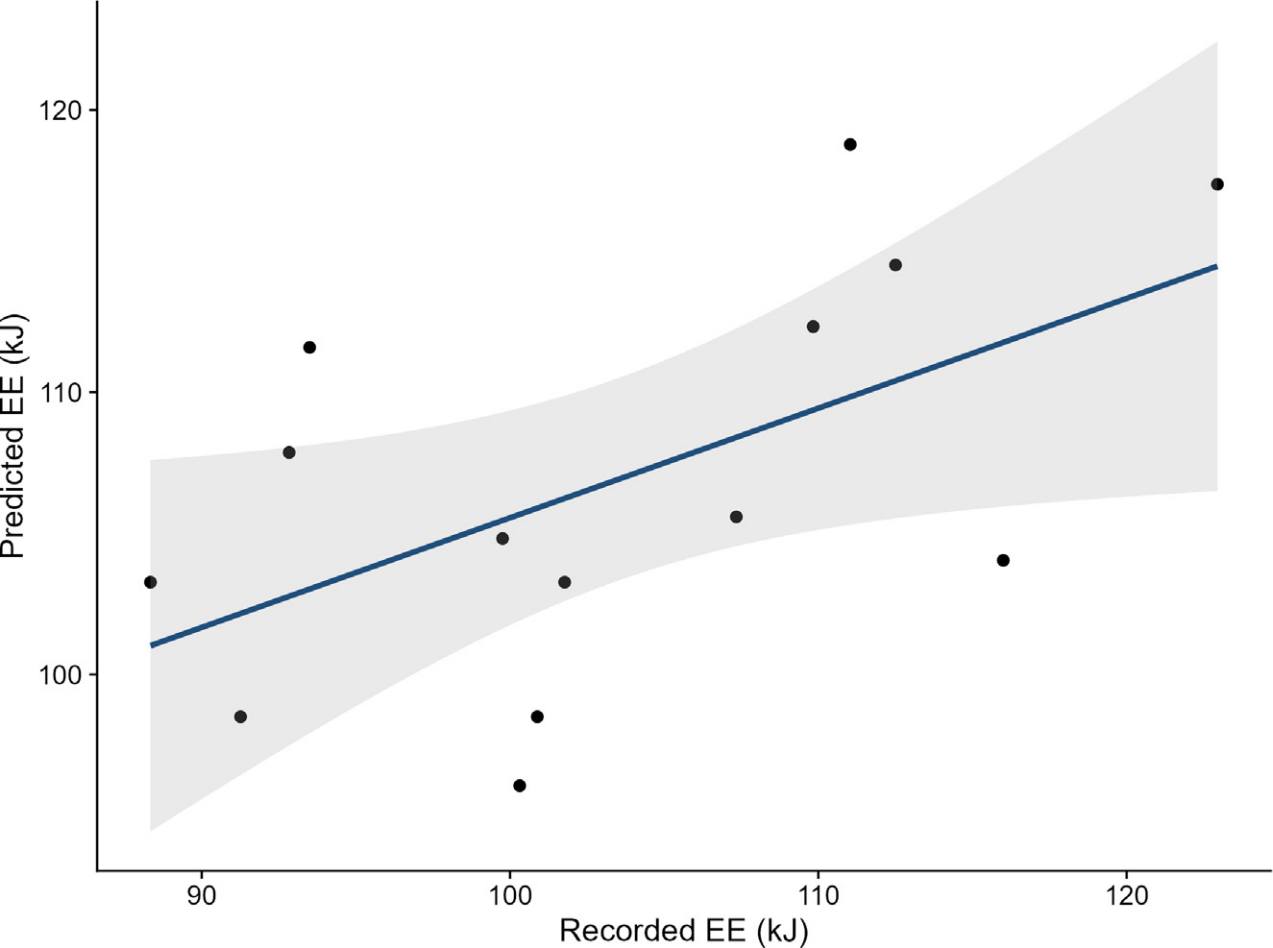
Relationship between predicted and recorded energy expenditure (EE; kJ) over a 73-h period Ptiloerection was allowed to increase fully, with feather depth equal to feather length. The line represents the relationship between the variables derived from a linear regression model with fat energy content as a covariate. The dots represent each individual, and the shaded area shows the 95% confidence interval.

**Figure 5:**
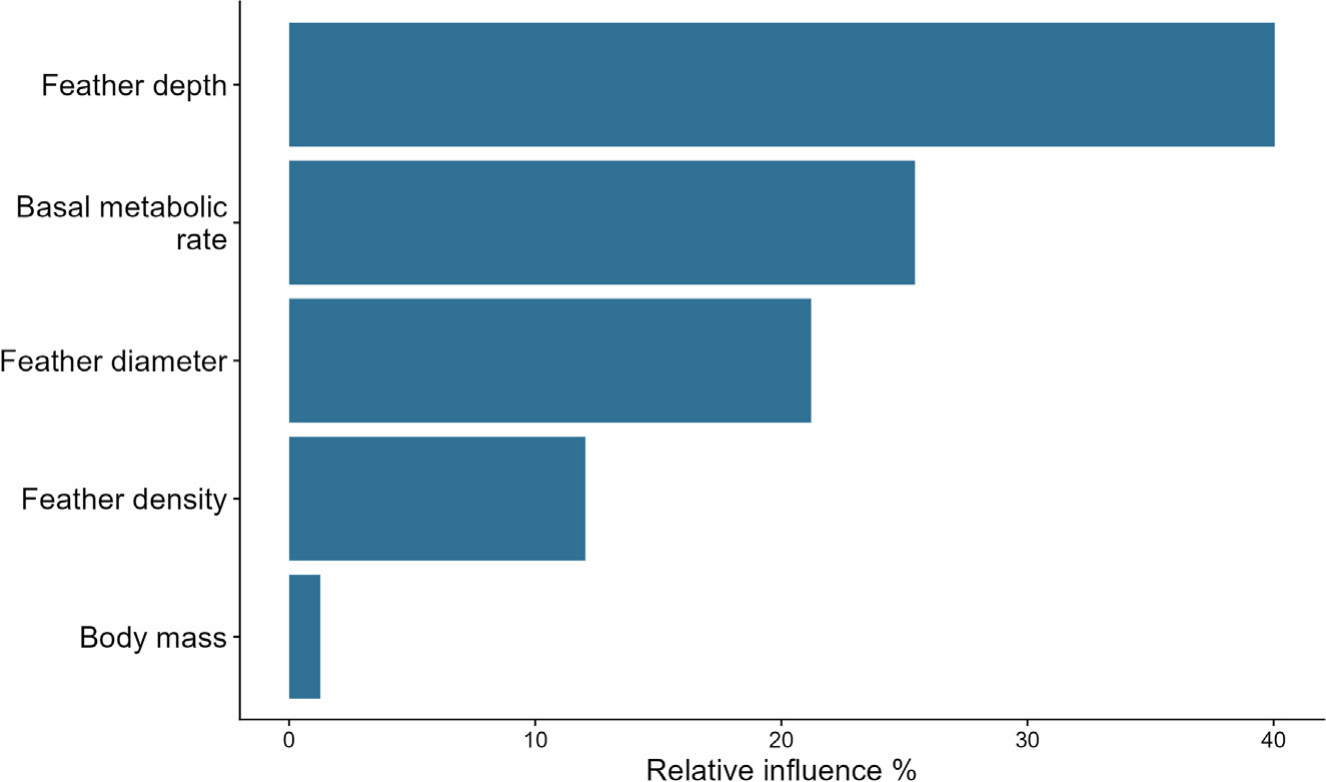
Relative influence of the most important parameters in predicting energy expenditure (EE) over a 73-h period

Mechanistic model predictions of hourly metabolic rate per individual for the 73 h are shown in Figure 3. The most precise match between the predicted and recorded EE was achieved when ptiloerection was allowed to fully increase, with feather depth equal to feather length. This scenario exhibited an RMSE of 9, outperforming the other scenarios with RMSE values of 11.5 (200% increase), 17.9 (75% increase), and 22.6 (35% increase) (Figure S2). For subsequent analyses, only the model incorporating full ptiloerection was considered. The mean EE prediction of this model was 106.89 ± 7.12 kJ (compared to the NEI estimates of 109.4 ± 15.5 kJ obtained in the bomb calorimetry experiments, see earlier text). Models testing for an association between modeled EE and observed NEI accounted for mass differences between the start and end of the experiment either by including the mass differences as a statistical covariate or by converting them to the energetic equivalents of mobilizing or storing fat. The best-ranked model was the one where predicted EE was obtained from NM with fat energy content as a covariate (Akaike information criterion [AIC] = 104.41; r = 0.97 ± 0.02, t-value = 39.43, d.f. = 12, r-square = 0.71) (Figure 4), followed by the NM model with mass change as a covariate (AIC = 114.81; r = 1.68 ± 0.39, t-value = 4.25, d.f. = 12, r-square = 0.38). Time-energy budget estimates of EE performed considerably worse compared to the NM estimates (AIC = 121.52 for the fat energy content model, AIC = 125.85 with mass as a covariate). The sensitivity analysis (Table 1) showed that predictions of EE in 73 h were largely determined by feather depth (variable importance 40%), followed by BMR (25.5%), feather diameter (21%), and feather density (12%) (Figure 5).

## Discussion

In this study, we evaluated the accuracy of a biophysical mechanistic model (NM) in predicting the TNZ and EE of common waxbills, small Afrotropical passerine birds that have successfully established invasive populations worldwide. Overall, our results show that the predicted TNZ and EE strongly correlated with empirically obtained TNZ through respirometry and with the energy intake obtained through bomb calorimetry of consumed food and excreta, respectively. For both predictions, different degrees of ptiloerection (200% and fully erect, respectively) exhibited the best performance. Despite the fact that ecophysiological models have been criticized as being data demanding, with as many as 60 different parameter values to be specified, the sensitivity of the models to parameter values has not yet been rigorously evaluated.^9^ In this study, we conducted an assessment of the sensitivity of NM to the parameterization of key biophysical traits associated with plumage thermal insulation capacity, metabolic rates, body mass, and body temperature. Our findings demonstrate that accurate estimation of species' TNZ and EE using NM is contingent upon precise parameterization of these traits. Thus, obtaining accurate data on these traits is crucial for developing reliable and robust models.

### Predicted versus observed metabolic rates along a temperature gradient

Pacioni et al.^52^ estimated the lower bound of common waxbill TNZ to be approximately 28°C, which closely aligns with our implementation of NM when increasing the feather depth through ptiloerection to three times the initial feather depth. This adjustment resulted in matching metabolic rates at different temperatures and a corresponding lower bound of the TNZ. It, however, remains difficult to *a priori* decide what degree of ptiloerection is ecologically most realistic as there is a dearth of information on the precise degree of ptiloerection in birds. The only study we are aware of is Hill et al.,^51^ who demonstrated that black-capped chickadees (*Poecile atricapillus*) exposed to progressively colder temperatures exhibited a ptiloerection that increased the depth of their feather layer with up to 35%. Two NM studies^37^^,^^38^ have proposed estimates of 75% and 200% for ptiloerection, but these values seem to be based on the optimal model fit of NM predictions and have not been thoroughly validated through empirical investigation. Our findings highlight the significance of considering the degree of ptiloerection in birds when using biophysical models such as NM. Consequently, further research dedicated to exploring to what extent birds can increase the depth of their feather layer through ptiloerection is crucial to gain a comprehensive understanding of the insulation benefits birds derive from this mechanism.

The sensitivity analyses conducted in our study reveal the importance of considering other feather characteristics in addition to ptiloerection, such as feather depth and density, which directly impact the insulation efficiency of birds (a thicker plumage layer corresponding to better insulation).^53^ Previous studies have shown that feather structure can vary within a species and is influenced by environmental conditions.^54^^,^^55^ For example, Barve et al.^56^ found that birds living in higher elevations and lower temperatures had an increase in the downy section of their feathers, and small birds had longer and more insulative feathers. In general, compared to birds living in warmer habitats, species residing in cold environments tend to have longer feathers^57^ that overlap and increase the feather depth.^56^ As it was difficult to accurately measure feather length and depth on live birds, all our feather measurements were taken from museum specimens from locations across the waxbills' native range. Import of wild birds into the European Union (EU) has been forbidden since 2005,^48^ so our common waxbills most likely stem from populations bred in captivity in Belgium since then. It is thus possible that our study population may have had different feather characteristics than birds in their native ranges, for example, growing longer feathers to increase insulation and limit heat loss. Thus, there are uncertainties associated with the feather depth of the actual measured population, while our simulations show that the degree of ptiloerection affects the results of our modeling.

While empirical information on waxbill metabolic rates at temperatures higher than 32°C is unavailable, even for the best fitting model (Figure 1C), observed metabolic rates at 32°C are at least somewhat higher than the BMR predicted by NM. This may be attributed to the challenge of accurately determining the true “basal” metabolic rates in birds as they tend to be sensitive to experimental conditions. For example, birds may experience being placed into metabolic chambers as stressful and not be completely at rest during measurements.^58^ When using the only available empirical estimate of ptiloerection (i.e., an increase in feather depth of 35%), NM tended to overpredict metabolic rates at temperatures below the waxbills’ TNZ (Figure 1A). This either means that the 35% estimate is incorrect for waxbills or could alternatively be explained by waxbills lowering their body temperature to conserve energy at colder temperatures (referred to as “heterothermy”). While some species of birds, particularly larger ones, are known to use heterothermy as an energy-saving mechanism, the extent to which this strategy is employed in small passerines such as waxbills is still not well understood.^59^ Recent studies, however, suggest that it may be more widespread in small passerines than previously thought.^59^^,^^60^ For example, Romano et al.^59^ found that superb fairy-wrens (*Malurus cyaneus*) significantly lowered their body temperature at night during mild winters, with variations of up to 10°C in their body temperature. This could suggest that during our TNZ experiments, common waxbills may have reduced their metabolic rates and conserved energy when faced with temperatures outside of their TNZ by lowering their body temperature. We currently lack the latter data to confirm or disprove whether common waxbills possess such a heterothermic ability. Further research on thermoregulation strategies in small passerines, including birds such as waxbills and estrildid finches, is thus needed to better understand the potential role of heterothermy in conserving energy in small passerines under varying ambient temperatures.

### Predicted versus observed EEs

The findings of our study indicate that mechanistic predictions of EE conform to empirically observed NEI values. The model that allowed feather depth to increase to the length of the feathers through full ptiloerection exhibited the best performance, closely followed by the model where feather depth was increased to three times the initial value (Figure S2). As during the food consumption experiment our study birds were exposed to temperatures ranging between 20°C and 22°C, it can be questioned how ecologically realistic the full ptiloerection scenario really is. First, while these temperatures are below the species TNZ, they do not represent very cold temperatures for waxbills (Pacioni et al.^52^) and visual observations during the experiment did not show the bird as fully fluffing their feathers. Second, and more conceptually, Morris^61^ noted that full ptiloerection may cause feathers to lose contact with each other, leading to a loss of insulation and facilitating cooling down in birds, instead of heat retention. Yet, independently from the ptiloerection scenario considered, our results demonstrate that NM outperforms time-energy budgets in predicting EE for small passerines. While time-energy budgets can serve as a first approximation of the daily EE of a species,^62^ such estimates often fall short when accounting for non-mechanical costs, such as those associated with thermoregulation.^27^^,^^63^^,^^64^ Although empirically measured metabolic rates were used to develop time-energy budgets in our study, inaccuracies in these predictions can likely be attributed to the fact that our study birds were kept at temperatures below their TNZ, requiring additional energy for thermoregulation that is not accounted for in the time-energy budget approach. In contrast, biophysical models such as NM are specifically designed to model such thermoregulatory costs, allowing for more accurate estimates of EE.

### Key biophysical traits

Sensitivity analyses are a valuable tool for identifying traits that have the strongest impact on predicted model estimates.^65^ Even though biophysical models require a considerable amount of trait data, our results suggest that only the most influential traits on model outcomes need detailed taxon-specific information. Here, our results indicate that certain key parameters, such as BMR, feather characteristics, specifically feather depth and density, body temperature, and body shape are essential for understanding thermal energetics in birds. Despite BMR being measured for many birds across different taxa (∼1,000 species),^66^ it still only represents a small fraction (∼10%) of the world’s bird species, and many of these measurements have only been taken once on a single individual,^58^ leaving substantial uncertainty regarding the metabolic information of several bird taxa. Although detailed datasets of trait data exist,^67^^,^^68^^,^^69^^,^^70^ several of the traits needed to make biophysical models widely applicable, particularly those related to feather characteristics, are not included.

### Conclusions and limitations of the study

Overall, the results of this study confirm that NM can provide good estimates of EE and TNZ of small passerine birds, such as common waxbills, provided that accurate measurements (or estimates) of key traits such as metabolic rates, body mass, body temperature, and plumage characteristics are available. When informed by accurate data on key traits, biophysical models may be used as a cost-efficient technique for assessing EE, and sensitivity analyses may be employed to identify those traits that may be most important for how species respond to changing environments, such as those caused by climate change or changes in land cover (i.e., deforestation-driven changes in thermal microclimates^71^).

Our results thus suggest that biophysical models could be used as a tool to better understand the potential expansion of this species in areas where it has been introduced. To achieve such forecasts, well-parameterized biophysical models do need to be combined with spatially explicit information on microclimates, food availability, water requirements, and responses to high temperature, as well as ontogenetic changes in these processes (including nesting). Thus, more research is needed to ensure the generalizability and accuracy of biophysical models across different contexts and species.

A limitation of our study is that the determination of the TNZ for common waxbills was based on a small sample size, which may introduce some uncertainty. Additionally, the lack of information regarding true body core temperature is another drawback of this study. However, it is worth noting that in small animals such as the common waxbill, changes in peripheral temperature are generally thought to correlate closely with core temperature.^72^^,^^73^ Future studies with larger sample sizes and direct measurements of core temperature would contribute to a more comprehensive understanding and further validate the findings of this research.

## STAR★Methods

### Key resources table

**Table:** 

REAGENT or RESOURCE	SOURCE	IDENTIFIER
**Biological samples**		

Bird excreta	Captive common waxbills	N/A

**Deposited data**		

Raw data and code	This paper	Mendeley Data: https://doi.org/10.17632/52z24w5jmb.2

**Experimental models: Organisms/strains**		

Common waxbill (*Estrilda astrild*), live birds	Commercial supplier	N/A
Common waxbill (*Estrilda astrild),* museum specimens collected across the species' native range	Koninklijk Belgisch Instituut voor Natuurwetenschappen, KBIN; Koninklijk Museum voor Midden-Afrika, KMMA; Muséum national d'Histoire naturelle, MNHN	Mendeley Data: https://doi.org/10.17632/52z24w5jmb.2

**Software and algorithms**		

R (version 4.3.0)	R Core Team	https://www.Rproject.org/
ExpeData (version 1.9.27)	Sable Systems	https://www.sablesys.com/

### Resource availability

#### Lead contact

For additional information and resource inquiries, please direct your requests to the lead contact, Marina Sentís Vila (marina.sentisvila@ugent.be).

#### Materials availability

Apart from generating data and R codes, this study did not produce any novel reagents or materials.

### Experimental model and study participant details

Metabolic rates and bird excreta were obtained from 14 captive adult common waxbills (*Estrilda astrild*). The sex of the birds was not included in this study because waxbills, especially outside of the breeding season, tend to be sexually monomorphic, and their sexes cannot be reliably identified based on plumage characteristics alone. All birds used in this study were obtained from a commercial supplier and kept in an outdoor aviary enriched with bamboo from late March 2021 to late October 2021. Birds were sheltered from direct rain and wind and had access to an indoor resting chamber where the temperature was kept above 10°C at all times (though birds rarely made use of it). Ethical Committee approval was obtained for this study from the Ethical Committee VIB/UGent - Faculty of Science, with reference code EC2021-055.

### Method details

#### Respirometry measurements

An open flow-through respirometry system that measures O_2_ consumption (VO_2_) and CO_2_ production (VCO_2_) was used, following Pacioni et al.^52^ To obtain daytime (resting) metabolic rates (RMR), 12 common waxbills were first fasted for 2 hours before respirometry, and they were then weighed to the nearest 0.1 g. Birds were placed in airtight plastic chambers (1.1 l), where ambient air was supplied by two pumps, and birds were measured in 10-minute cycles for a total of 3 cycles during the day (∼4 hours). Birds were kept in the metabolic chambers from 13.30 h to 17.30 h and exposed to natural daylight. All chambers were kept inside a customised climate control unit (Combisteel R600) set to ambient temperatures (20.0°C – 21.5°C). The supplied ambient air was divided into 8 streams and directed to a mass-flow meter (FB-8, Sable Systems), with a flow of 650 ml/min. From the flow meter, the airstreams were directed to the 8 metabolic chambers (one baselining chamber, and seven chambers containing birds). The excurrent airstreams from the chambers were connected to a multiplexer (RM-8, Sable Systems), allowing each chamber to be sampled independently from the others. Air from the birds and baseline channels were subsampled and pulled through a Field Metabolic System (FMS-3, Sable Systems). After the measurements, waxbills were weighed again to the nearest 0.1 g and were fed *ad libitum*. The software ExpeData (Sable Systems) was used to record trials and extract VO_2_ (ml/min) using equation 9.7 from Lighton.^74^

Basal metabolic rates (BMR) and thermoneutral zones (TNZ) were taken from Pacioni et al.^52^ To obtain the common waxbill’s TNZ, Pacioni et al.^52^ measured VO_2_ of three post-absorptive individuals at a series of different air temperatures at night. The temperatures were randomly selected during the night from a set of 9 temperatures that ranged from 15°C to 32°C. BMR of the 14 individuals used here was measured at night, using air temperatures of 28°C. Similar to RMR measurements, the birds were fasted and enclosed in airtight plastic chambers, with an airflow set at 650 ml/min. Cycles of measurements alternated between birds and several baseline measurements, with the duration and timing of each bird's measurement depending on the number of birds per session (usually around 30 minutes per bird, involving three cycles during the night). These measurements lasted for an average duration of 9 hours. Body temperatures were also taken from Pacioni et al.,^52^ who used a T-type thermocouple (5SC-TT-TI-36-2M, Omega) attached to the skin over the pectoral muscles of the birds during measurements of summit metabolism.

#### Morphological measurements

Because common waxbills are very small, active birds, several morphological variables required by NM could not be accurately measured on live birds. Therefore, the following morphological variables were measured instead on 340 common waxbill specimens from across the species’ native range held at three natural history museums (KBIN, Brussels; KMMA, Tervuren; MNHN, Paris). Feather characteristics such as feather depth and length were measured to the nearest 0.01 mm dorsally and ventrally, twice at the same spot, for the torso of the specimens using a blunt probe. For each feather trait, the mean between the two values was obtained. For feather depth, the probe was inserted perpendicularly to the skin surface, with the probe touching the skin. The probe was marked where it visually disappeared into the feathers. To obtain feather length, the probe was inserted parallel to the feathers. Once it touched the skin and base of the feather, the probe was marked where it disappeared into the feathers. Feather depths and lengths were then obtained by measuring the distance between the mark and the tip of the probe using a digital calliper. Feather depth estimates were on average (estimate and sd) 2.2 ± 0.8 mm dorsally and 2.5 ± 0.9 mm ventrally, and feather length was 16.1 ± 2.5 mm dorsally and 14.1 ± 2.3 mm ventrally. Dorsal and ventral feather density values were set to 50 1/mm^2^.^75^ The birds’ body shape was modelled as an ellipsoid, and the maximum ratio of length to width was set to 3 (Figure S1). Body temperature increments were set to 0.25°C,^75^ the percentage of body fat to 4%,^76^ and body density to 875 kg/m^3^.^42^^,^^77^ Feather thermal conductivity was set to 0.209 W/mK (www.engineeringtoolbox.com), and the fractional depth of fur at which longwave radiation is exchanged was set to 0.9.^37^ Percentage of wet skin was set to 1% ^38^). Offset between air temperature and breath was set to 5°C, and O_2_ extraction efficiency was set to 25%.^75^ Maximum panting rate and the multiplier on BMR at maximum panting level were set to 15 and 1, respectively.^75^ All other NM parameter estimates were left at their default values.

#### Energy expenditure (EE) experiments

During October 2021, the 14 waxbills were placed individually in small cages (35 cm x 29 cm x 23 cm) where they could maintain visual and auditory contact with each other. Cages were laterally covered by transparent plastic to avoid food spilling. During the trials, birds were given water and 50.0 g of hulled millet, *Panicum miliaceum* (15.66 MJ/kg and 11% crude protein) for 73 h. Prior to the experiments, the birds were fasted for three hours and weighed to the nearest 0.1 g. After 73 h, food was removed for another three hours and, afterwards, birds were weighed again and placed back in the aviary. Ambient temperatures were recorded using a T-type thermocouple (5SC-TT-TI-36-2M, Omega). Excreta and food leftovers were collected and weighed with an electronic analytical balance (± 0.0001 g). Excreta and food samples were lyophilized, weighed and homogenised in a mill.

The gross energy content of the food and each individual’s food leftovers and excreta was determined in a bomb calorimeter. The net energy intake (NEI) was estimated by first calculating the metabolizable energy intake, which is defined as the difference in calories between the food ingested (food offered minus food leftover) and the calories in the excreta. To account for the energetic cost of digestion, we derived a coefficient of 0.83 from Meienberger & Dauberschmidt^78^ and multiplied our metabolizable energy intake values with this coefficient to obtain our NEI values. As NEI can be considered equal to EE^50^ only when animals maintain constant body mass, we took the difference in body mass at the start and end of the 73-h experimental period into account by a) including mass difference as a fixed effect covariate in the statistical analysis (see below), and b) converting mass changes to energy either stored or mobilised from fat. Assuming that changes in body mass primarily reflect either the mobilisation of energy reserves from fat (when waxbills lost mass) or energy storage as fat (when birds gained weight), we used an estimate of 39.5 kJ energy that can be mobilised from 1 g of fat^79^ and a relative metabolic cost of storing fat of 1.17 kJ expended per 1 kJ stored as fat.^80^

#### Thermoneutral zone (TNZ) predictions

To test NM predictions of the common waxbill’s TNZ against the empirical TNZ measurements of Pacioni et al.,^52^ NM was parameterised based on the key species traits mentioned above. For all the predictions, NicheMapR endotherm model ‘version 3.1’^75^ was used. For BMR and body masses, individual-level measurements were taken from Pacioni et al.,^52^ using the measurements obtained in the summer. A body temperature of 39.1°C was used at night and 41.6°C at daytime, obtained from Pacioni et al.,^52^ as small birds (∼10 g) at night typically have a body temperature that is about ∼2.48°C lower than during the day.^81^ Here, to account for changes in thermal conductance caused by ptiloerection, we performed a scenario-based approach where we allowed feather depth to increase by 35%,^51^ 75%,^37^ 200%^38^ and full ptiloerection, where feather depth equalled feather length. At each ambient air temperature, we selected the degree of ptiloerection that resulted in the lowest metabolic rate. In the version of NicheMapR that we used, ptiloerection was manually coded (see Deposited Data). However, the latest release of NicheMapR now enables the specification of the maximum feather depth, with the option to gradually reduce it using the 'TREGMODE' code argument (github.com/mrke/NicheMapR). To simulate metabolic chamber conditions, wind speed was set to 0.1 m/s, relative humidity to 5%, and solar radiation to 0.^22^ Air temperatures were set to gradually increase from 10°C to 40°C with 1°C increment. Pacioni et al.^52^ did not record metabolic rates above 32°C, allowing to only derive the lower bound of common waxbill’s TNZ. Therefore, we here only evaluate NM capacity to identify the lower TNZ bound.

#### Energy expenditure (EE) predictions

NicheMapper models hourly metabolic heat production (W) required for the individual to remain in homeothermy. These estimates can then be used to calculate the total EE for a specific time period. To parameterize NM for estimating EE, we applied the same morphological and physiological traits used for TNZ predictions, except for BMR and body masses. Instead, individual-level measurements obtained in autumn from Pacioni et al.^52^ were used. For the RMR, the metabolic rates could only be obtained for 12 of the common waxbills, so the mean RMR was used for the remaining two birds. A model that differentiates activity levels was run, where RMR values and daytime body temperatures were used during the day and BMR values and night-time body temperatures during the night. During the night, ptiloerection was accounted for as above, with the four scenarios. Recorded hourly ambient temperatures during the 73-h experiments (19.6°C - 22.3°C) were fed to NM to obtain predictions of hourly waxbill metabolic heat production, which was then summed to obtain the total EE of waxbills for 73 h, and finally converted to kilocalories. These predictions were compared with the EE obtained from the 14 experimental common waxbills.

Daily EE can be determined by multiplying the time spent on various activities by their associated energy costs.^27^ For this study, the predicted EE using time-energy budgets was based on the assumption that the birds were resting but not sleeping, as they were in a small cage and unable to fly much. Therefore, following Fitzpatrick et al.,^42^ an activity multiplier of 2 times the BMR was used. During the 73-h experiment, the total duration of daylight hours was 40 so the BMR and the activity multiplier were thus multiplied by this duration. For the remaining 33 hours, it was assumed that the birds were sleeping, so the night-time hours were multiplied by the birds' BMR to estimate their energy expenditure during the sleeping period. The total EE used in 73 h was obtained by adding both values.

#### Sensitivity analyses

To determine which parameters most strongly influenced NM predictions of TNZ and EE, a sensitivity analysis was performed using 9 of the above-mentioned morphological and physiological parameters (Table 1). These variables were added to a Latin Hypercube (LHS) that varied estimates between the minimum and maximum values obtained from the waxbill measurements and literature surveys. Thousand model variants were obtained. Sensitivity analyses for TNZ (simulated from 10°C to 40°C) and EE (using the empirical 73-hour ambient temperatures) were run using the same NM setting and procedures as described above.

### Quantification and statistical analysis

To compare the performance of the different NM models when predicting TNZ and EE with different degrees of ptiloerection, the Root Mean Square Error (RMSE) was used. For EE predictions, the model with the lowest (best) RMSE was selected for further analyses. Four linear models were then employed, and their intercepts were forced through zero. Two of the models compared the observed EE with the predicted EE obtained from NM, with one model incorporating mass change as a covariate, and the other model utilising the energy required for mobilising or storing fat (see above) as a covariate. The other two models compared the observed EE with the predicted EE derived from time-energy budgets, again, with one model using mass change as a covariate, and the other model using the energy required for mobilising or storing fat as a covariate. The best fitting model was selected based on the lowest Akaike information criterion (AIC) score. AIC determines which competing model most closely approximates the real mechanism underlying the biological event under study by comparing and ranking multiple models.^83^

To investigate which values influenced most strongly NM outcomes of TNZ and EE predictions, a generalized boosted regression model (GBM) was applied to the model variants obtained from the LHS using the gbm function in the ‘gbm’ R package.^84^ The impact of the 9 parameters on the output variables was estimated and was used to quantitatively measure their relative influence. The output variables were the EE in 73 h, and the lower bound of the predicted TNZ. The EE was obtained as the sum of predicted hourly metabolic rates, for 73 h. The lower bound of the predicted TNZ, which is the point at which the metabolic rate curve stops decreasing and starts to plateau, was obtained by identifying the inflexion point of the metabolic rate curve using the second derivative.

## Data Availability

• The raw data underlying this study have been deposited at Mendeley Data and are publicly available as of the date of publication. DOI is listed in the key resources table. • All original code has been deposited at Mendeley Data and is publicly available as of the date of publication. DOI is listed in the key resources table. • Any additional information required to reanalyze the data reported in this paper is available from the lead contact upon request.

## References

[1] McCarty J.P. (2001). Ecological consequences of recent climate change. Conserv. Biol..

[2] Diez J.M., D’Antonio C.M., Dukes J.S., Grosholz E.D., Olden J.D., Sorte C.J., Blumenthal D.M., Bradley B.A., Early R., Ibáñez I. (2012). Will extreme climatic events facilitate biological invasions?. Front. Ecol. Environ..

[3] Early R., Bradley B.A., Dukes J.S., Lawler J.J., Olden J.D., Blumenthal D.M., Gonzalez P., Grosholz E.D., Ibañez I., Miller L.P. (2016). Global threats from invasive alien species in the twenty-first century and national response capacities. Nat. Commun..

[4] Urban M.C. (2015). Climate change. Accelerating extinction risk from climate change. Science.

[5] Pyšek P., Hulme P.E., Simberloff D., Bacher S., Blackburn T.M., Carlton J.T., Dawson W., Essl F., Foxcroft L.C., Genovesi P. (2020). Scientists’ warning on invasive alien species. Biol. Rev. Camb. Phil. Soc..

[6] Bergström U., Sundblad G., Downie A.L., Snickars M., Boström C., Lindegarth M. (2013). Evaluating eutrophication management scenarios in the Baltic Sea using species distribution modelling. J. Appl. Ecol..

[7] Guisan A., Tingley R., Baumgartner J.B., Naujokaitis-Lewis I., Sutcliffe P.R., Tulloch A.I.T., Regan T.J., Brotons L., McDonald-Madden E., Mantyka-Pringle C. (2013). Predicting species distributions for conservation decisions. Ecol. Lett..

[8] Urban M.C., Bocedi G., Hendry A.P., Mihoub J.B., Pe’er G., Singer A., Bridle J.R., Crozier L.G., De Meester L., Godsoe W. (2016). Improving the forecast for biodiversity under climate change. Science.

[9] Peterson A.T., Papeş M., Soberón J. (2015).

[10] Kearney M., Simpson S.J., Raubenheimer D., Helmuth B. (2010). Modelling the ecological niche from functional traits. Philos. Trans. R. Soc. Lond. B Biol. Sci..

[11] Araújo M.B., Anderson R.P., Márcia Barbosa A., Beale C.M., Dormann C.F., Early R., Garcia R.A., Guisan A., Maiorano L., Naimi B. (2019). Standards for distribution models in biodiversity assessments. Sci. Adv..

[12] Guillera-Arroita G., Lahoz-Monfort J.J., Elith J., Gordon A., Kujala H., Lentini P.E., McCarthy M.A., Tingley R., Wintle B.A. (2015). Is my species distribution model fit for purpose? Matching data and models to applications. Global Ecol. Biogeogr..

[13] Elith J., Leathwick J.R. (2009). Species Distribution Models: Ecological Explanation and Prediction Across Space and Time. Annu. Rev. Ecol. Evol. Syst..

[14] Helmuth B., Kingsolver J.G., Carrington E. (2005). Biophysics, physiological ecology, and climate change: does mechanism matter?. Annu. Rev. Physiol..

[15] Buckley L.B., Urban M.C., Angilletta M.J., Crozier L.G., Rissler L.J., Sears M.W. (2010). Can mechanism inform species’ distribution models?. Ecol. Lett..

[16] Santini L., Benítez-López A., Maiorano L., Čengić M., Huijbregts M.A.J. (2021). Assessing the reliability of species distribution projections in climate change research. Divers. Distrib..

[17] Liu C., Wolter C., Xian W., Jeschke J.M. (2020). Species distribution models have limited spatial transferability for invasive species. Ecol. Lett..

[18] Fuller A., Dawson T., Helmuth B., Hetem R.S., Mitchell D., Maloney S.K. (2010). Physiological mechanisms in coping with climate change. Physiol. Biochem. Zool..

[19] Teal L.R., Marras S., Peck M.A., Domenici P. (2018). Physiology-based modelling approaches to characterize fish habitat suitability: Their usefulness and limitations. Estuar. Coast Shelf Sci..

[20] Tourinho L., Vale M.M. (2023). Choosing among correlative, mechanistic, and hybrid models of species’ niche and distribution. Integr. Zool..

[21] Porter W.P., Ostrowski S., Williams J.B. (2010). Modeling animal landscapes. Physiol. Biochem. Zool..

[22] Mathewson P.D., Porter W.P., Barrett L., Fuller A., Henzi S.P., Hetem R.S., Young C., McFarland R. (2020). Field data confirm the ability of a biophysical model to predict wild primate body temperature. J. Therm. Biol..

[23] Kearney M.R., Jusup M., McGeoch M.A., Kooijman S.A.L.M., Chown S.L. (2021). Where do functional traits come from? The role of theory and models. Funct. Ecol..

[24] Briscoe N.J., Morris S.D., Mathewson P.D., Buckley L.B., Jusup M., Levy O., Maclean I.M.D., Pincebourde S., Riddell E.A., Roberts J.A. (2023). Mechanistic Forecasts of Species Responses to Climate Change: The Promise of Biophysical Ecology. Global Change Biol..

[25] Mathewson P.D., Moyer-Horner L., Beever E.A., Briscoe N.J., Kearney M., Yahn J.M., Porter W.P. (2017). Mechanistic variables can enhance predictive models of endotherm distributions: the American pika under current, past, and future climates. Global Change Biol..

[26] Kearney M., Porter W. (2009). Mechanistic niche modelling: combining physiological and spatial data to predict species’ ranges. Ecol. Lett..

[27] Goldstein D.L. (1988). Estimates of daily energy expenditure in birds: The time-energy budget as an integrator of laboratory and field studies. Am. Zool..

[28] Kronfeld-Schor N., Dayan T. (2013). Thermal ecology, environments, communities, and global change: Energy intake and expenditure in endotherms. Annu. Rev. Ecol. Evol. Syst..

[29] Khaliq I., Hof C., Prinzinger R., Böhning-Gaese K., Pfenninger M. (2014). Global variation in thermal tolerances and vulnerability of endotherms to climate change. Proc. Biol. Sci..

[30] Oswald S.A., Huntley B., Collingham Y.C., Russell D.J.F., Anderson B.J., Arnold J.M., Furness R.W., Hamer K.C. (2011). Physiological effects of climate on distributions of endothermic species. J. Biogeogr..

[31] Fuller A., Tran T., Muhumuza M., Haglund M.M. (2016). Towards a mechanistic understanding of the responses of large terrestrial mammals to heat and aridity associated with climate change. Clim. Change Responses.

[32] Grunst M.L., Grunst A.S., Grémillet D., Kato A., Bustamante P., Albert C., Brisson-Curadeau É., Clairbaux M., Cruz-Flores M., Gentès S. (2023). A keystone avian predator faces elevated energy expenditure in a warming Arctic. Ecology.

[33] McKechnie A.E., Gerson A.R., Wolf B.O. (2021). Thermoregulation in desert birds: scaling and phylogenetic variation in heat tolerance and evaporative cooling. J. Exp. Biol..

[34] Kronfeld-Schor N. (2014). Conservation physiology: A new challenge for thermal biologists. Temperature.

[35] Pilowsky J.A., Colwell R.K., Rahbek C., Fordham D.A. (2022). Process-explicit models reveal the structure and dynamics of biodiversity patterns. Sci. Adv..

[36] Kearney M.R., Porter W.P., Murphy S.A. (2016). An estimate of the water budget for the endangered night parrot of Australia under recent and future climates. Clim. Chang. Responses.

[37] Porter W.P., Budaraju S., Stewart W.E., Ramankutty N. (2000). Calculating Climate Effects on Birds and Mammals: Impacts on Biodiversity, Conservation, Population Parameters, and Global Community Structure. Am. Zool..

[38] Porter W.P., Vakharia N., Klousie W.D., Duffy D. (2006). Po’ouli landscape bioinformatics models predict energetics, behavior, diets, and distribution on Maui. Integr. Comp. Biol..

[39] Fort J., Porter W.P., Grémillet D. (2009). Thermodynamic modelling predicts energetic bottleneck for seabirds wintering in the northwest Atlantic. J. Exp. Biol..

[40] Mathewson P.D., Hanson-Dorr K.C., Porter W.P., Bursian S.J., Dean K.M., Healy K., Horak K., Link J.E., Harr K.E., Dorr B.S. (2018). Experimental and modeled thermoregulatory costs of repeated sublethal oil exposure in the Double-crested Cormorant. Mar. Pollut. Bull..

[41] Göktepe Ö., Hundt P., Porter W., Pereira D. (2012). Comparing bioenergetics models of double-crested cormorant (*Phalacrocorax auritus*) fish consumption. Waterbirds.

[42] Fitzpatrick M.J., Mathewson P.D., Porter W.P. (2015). Validation of a Mechanistic Model for Non-Invasive Study of Ecological Energetics in an Endangered Wading Bird with Counter-Current Heat Exchange in its Legs. PLoS One.

[43] Conradie S.R., Kearney M.R., Wolf B.O., Cunningham S.J., Freeman M.T., Kemp R., McKechnie A.E. (2023). An evaluation of a biophysical model for predicting avian thermoregulation in the heat. J. Exp. Biol..

[44] Speakman J.R., Thomas D.W., Kunz T.H., Fenton M.B. (2003). Bat ecology.

[45] Blackburn T.M., Gaston K.J. (1994). The Distribution of Body Sizes of the World’s Bird Species. Oikos.

[46] Cardoso G.C., Reino L., Queiroz A.I., Pooley S. (2018). Histories of Bioinvasions in the Mediterranean.

[47] Stiels D., Schidelko K., Engler J.O., van den Elzen R., Rödder D. (2011). Predicting the potential distribution of the invasive Common Waxbill *Estrilda astrild* (Passeriformes: Estrildidae). J. Ornithol..

[48] Reino L., Figueira R., Beja P., Araújo M.B., Capinha C., Strubbe D. (2017). Networks of global bird invasion altered by regional trade ban. Sci. Adv..

[49] Dyer E.E., Redding D.W., Blackburn T.M. (2017). The global avian invasions atlas, a database of alien bird distributions worldwide. Sci. Data.

[50] Lichtenbelt W.D.V.M., Wesselingh R.A., Vogel J.T., Albers K.B.M. (1993). Energy budgets in free-living green iguanas in a seasonal environment. Ecology.

[51] Hill R.W., Beaver D.L., Veghte J.H. (1980). Body Surface Temperatures and Thermoregulation in the Black-Capped Chickadee (*Parus atricapillus*). Physiol. Zool..

[52] Pacioni C., Sentís M., Kerimov A., Bushuev A., Lens L., Strubbe D. (2023). Seasonal variation in thermoregulatory capacity of three closely related Afrotropical Estrildid finches introduced to Europe. J. Therm. Biol..

[53] McCafferty D.J., Pandraud G., Gilles J., Fabra-Puchol M., Henry P.Y. (2017). Animal thermoregulation: a review of insulation, physiology and behaviour relevant to temperature control in buildings. Bioinspiration Biomimetics.

[54] Broggi J., Gamero A., Hohtola E., Orell M., Nilsson J.Å. (2011). Interpopulation variation in contour feather structure is environmentally determined in great tits. PLoS One.

[55] Cooper S.J. (2002). Seasonal metabolic acclimatization in mountain chickadees and juniper titmice. Physiol. Biochem. Zool..

[56] Barve S., Ramesh V., Dotterer T.M., Dove C.J. (2021). Elevation and body size drive convergent variation in thermo-insulative feather structure of Himalayan birds. Ecography.

[57] Pap P.L., Vincze O., Wekerle B., Daubner T., Vágási C.I., Nudds R.L., Dyke G.J., Osváth G. (2017). A phylogenetic comparative analysis reveals correlations between body feather structure and habitat. Funct. Ecol..

[58] McKechnie A.E., Wolf B.O. (2004). The allometry of avian basal metabolic rate: good predictions need good data. Physiol. Biochem. Zool..

[59] Romano A.B., Hunt A., Welbergen J.A., Turbill C. (2019). Nocturnal torpor by superb fairy-wrens: a key mechanism for reducing winter daily energy expenditure. Biol. Lett..

[60] Geiser F. (2019). Frequent nocturnal torpor in a free-ranging Australian honeyeater, the noisy miner. Naturwissenschaften.

[61] Morris D. (1956). The Feather Postures of Birds and the Problem of the Origin of Social Signals. Beyond Behav..

[62] Fort J., Porter W.P., Grémillet D. (2011). Energetic modelling: a comparison of the different approaches used in seabirds. Comp. Biochem. Physiol. Mol. Integr. Physiol..

[63] Ste-Marie E., Grémillet D., Fort J., Patterson A., Brisson-Curadeau É., Clairbaux M., Perret S., Speakman J.R., Elliott K.H. (2022). Accelerating animal energetics: high dive costs in a small seabird disrupt the dynamic body acceleration-energy expenditure relationship. J. Exp. Biol..

[64] Dickinson E.R., Stephens P.A., Marks N.J., Wilson R.P., Scantlebury D.M. (2021). Behaviour, temperature and terrain slope impact estimates of energy expenditure using oxygen and dynamic body acceleration. Anim. Biotelemetry.

[65] Augusiak J., Van den Brink P.J., Grimm V. (2014). Merging validation and evaluation of ecological models to ‘evaludation’: A review of terminology and a practical approach. Ecol. Modell..

[66] Gavrilov V.M., Golubeva T.B., Bushuev A.V. (2022). Evolution of metabolic scaling among the tetrapod: effect of phylogeny, the geologic time of class formation, and uniformity of species within a class. Integr. Zool..

[67] Tobias J.A., Sheard C., Pigot A.L., Devenish A.J.M., Yang J., Sayol F., Neate-Clegg M.H.C., Alioravainen N., Weeks T.L., Barber R.A. (2022). AVONET: morphological, ecological and geographical data for all birds. Ecol. Lett..

[68] Wilman H., Belmaker J., Simpson J., de la Rosa C., Rivadeneira M.M., Jetz W. (2014). EltonTraits 1.0: Species-level foraging attributes of the world’s birds and mammals. Ecology.

[69] Bennett J.M., Calosi P., Clusella-Trullas S., Martínez B., Sunday J., Algar A.C., Araújo M.B., Hawkins B.A., Keith S., Kühn I. (2018). GlobTherm, a global database on thermal tolerances for aquatic and terrestrial organisms. Sci. Data.

[70] Marques G.M., Augustine S., Lika K., Pecquerie L., Domingos T., Kooijman S.A.L.M. (2018). The AmP project: Comparing species on the basis of dynamic energy budget parameters. PLoS Comput. Biol..

[71] Ewers R.M., Banks-Leite C. (2013). Fragmentation impairs the microclimate buffering effect of tropical forests. PLoS One.

[72] Wacker C.B., Daniella Rojas A., Geiser F. (2012). The use of small subcutaneous transponders for quantifying thermal biology and torpor in small mammals. J. Therm. Biol..

[73] McCafferty D.J., Gallon S., Nord A. (2015). Challenges of measuring body temperatures of free-ranging birds and mammals. Anim. Biotelemetry.

[74] Lighton J.R.B. (2018).

[75] Kearney M.R., Briscoe N.J., Mathewson P.D., Porter W.P. (2021). NicheMapR – an R package for biophysical modelling: the endotherm model. Ecography.

[76] Ward P. (1969). Seasonal and Diurnal Changes in the Fat Content of an Equatorial Bird. Physiol. Zool..

[77] Hamershock D.W., Seamans T.W., Bernhardt G.E. (1993).

[78] Meienberger C., Dauberschmidt C. (1992). Kann die ”spezifisch dynamische Wirkung“ einen Beitrag zur Thermoregulation körnerfressender Singvögel leisten?. J. Ornithol..

[79] Molokwu M.N., Nilsson J.Å., Olsson O. (2011). Diet selection in birds: trade-off between energetic content and digestibility of seeds. Behav. Ecol..

[80] Roberts S.B., Young V.R. (1988). Energy costs of fat and protein deposition in the human infant. Am. J. Clin. Nutr..

[81] Prinzinger R., Preßmar A., Schleucher E. (1991). Body temperature in birds. Comp. Biochem. Physiol. A Physiol..

[82] Deville A.S., Labaude S., Robin J.P., Béchet A., Gauthier-Clerc M., Porter W., Fitzpatrick M., Mathewson P., Grémillet D. (2014). Impacts of extreme climatic events on the energetics of long-lived vertebrates: the case of the greater flamingo facing cold spells in the Camargue. J. Exp. Biol..

[83] Symonds M.R.E., Moussalli A. (2011). A brief guide to model selection, multimodel inference and model averaging in behavioural ecology using Akaike’s information criterion. Behav. Ecol. Sociobiol..

[84] Greenwel B., Boehmke B., Cunningham J., Developers G.B.M. (2022).

