# Supplementary material for: Biophysical models accurately characterize the thermal energetics of a small invasive passerine bird
Source: iScience. 2023 Aug 26;26(10):107743. doi: 10.1016/j.isci.2023.107743 (PMC10504485; doi:10.1016/j.isci.2023.107743)
Supplement: Document S1. Figures S1 and S2 [file mmc1.pdf]

iScience, Volume *26*

Supplemental information

Biophysical models accurately characterize the thermal energetics of a small invasive passerine bird

Marina Sentís, Cesare Pacioni, Annelies De Cuyper, Geert P.J. Janssens, Luc Lens, and Diederik Strubbe

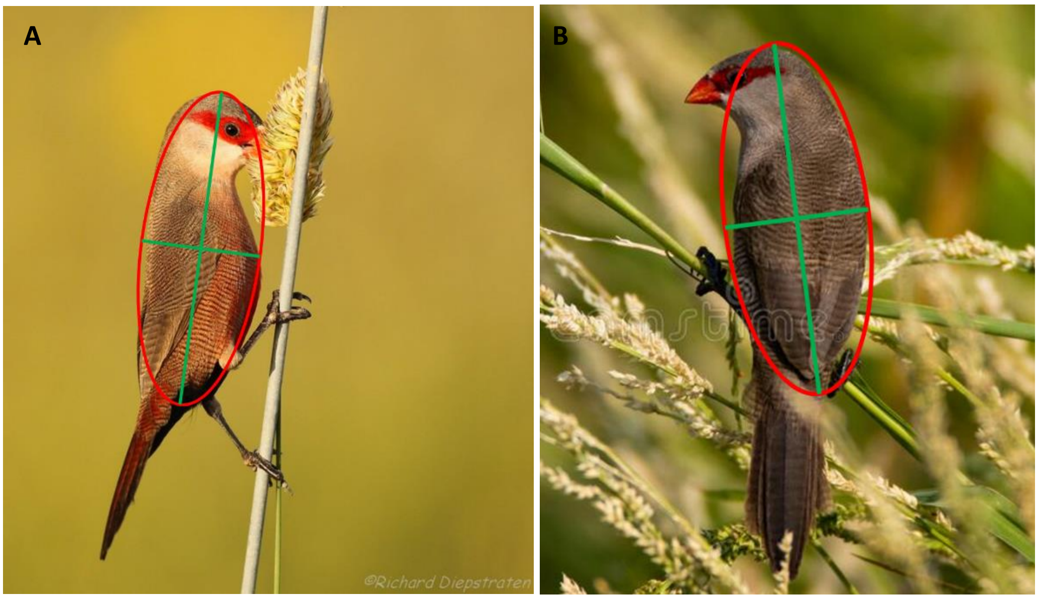

Figure S1. Examples of estimates of maximum ratio of length to width of common waxbills, related to Table 1 and STAR methods. A) 4,7/1,8 = 2,611 and B) 3.7/1.4 = 2.643.

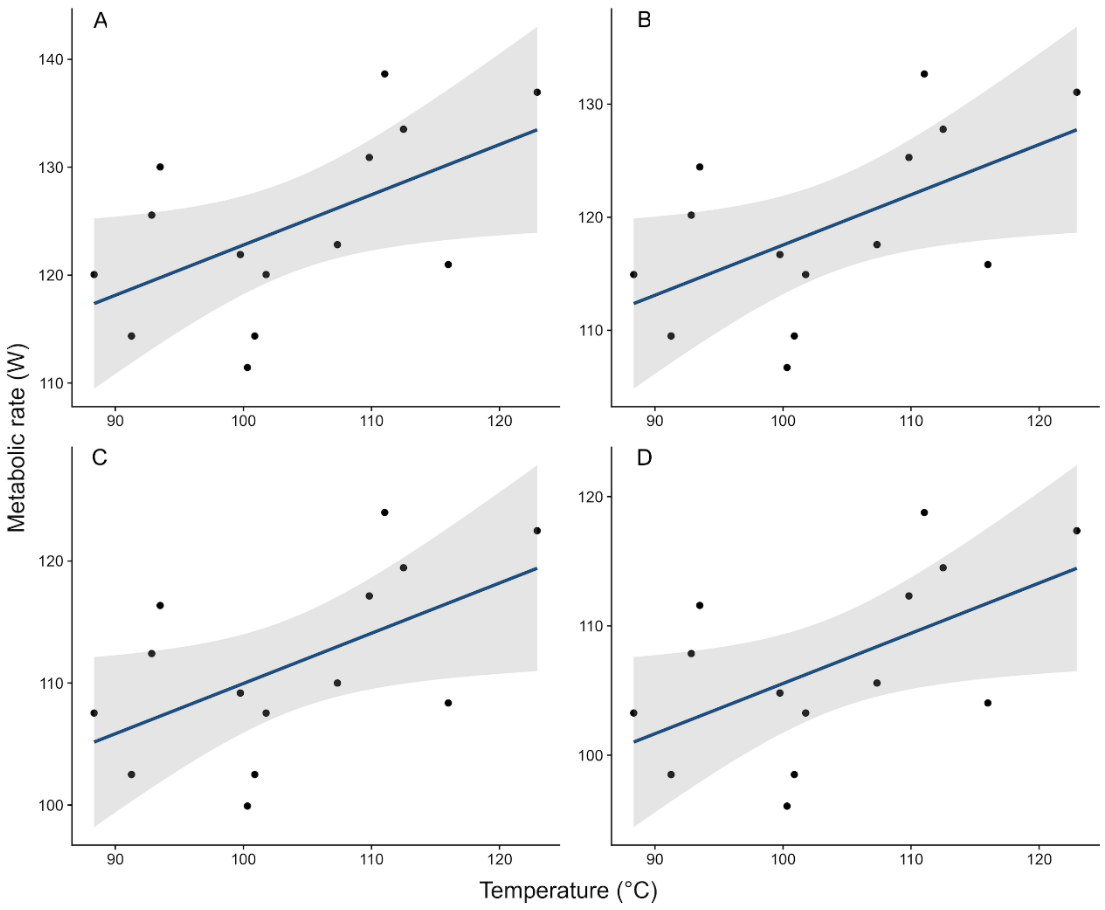

Figure S2. Relationship between predicted and recorded energy expenditure (EE; kJ) over a 73-hour using different degrees of ptiloerection, related to Fig. 4. A) Increase in feather depth of 35 %; B) increase in feather depth of 75 %; C) increase in feather depth of 200 %; D) full increase in feather depth, where feather depth equals feather length. The line represents the relationship between the variables derived from a linear regression model with fat energy content as a covariate. The dots represent each individual, and the shaded area shows the 95 % confidence interval.
